# Using donor funding to catalyse investment in malaria prevention in Ghana: an analysis of the potential impact on public and private sector expenditure

**DOI:** 10.1186/s12936-022-04218-2

**Published:** 2022-06-27

**Authors:** Lucy Paintain, Richard Kpabitey, Felix Nyanor-Fosu, Danielle Piccinini Black, Kathryn Bertram, Jayne Webster, Catherine Goodman, Matt Lynch

**Affiliations:** 1 Department of Disease Control, London School of Hygiene & Tropical Medicine, Keppel Street, London, WC1E 7HT UK; 2 Johns Hopkins Center for Communication Programs, 111 Market Place, Suite 310, Baltimore, MD 21202 USA; 3 Department of Global Health and Development, London School of Hygiene & Tropical Medicine, 15-17 Tavistock Place, London, WC1H 9SH UK

## Abstract

**Background:**

An estimated 1.5 billion malaria cases and 7.6 million malaria deaths have been averted globally since 2000; long-lasting insecticidal nets (LLINs) have contributed an estimated 68% of this reduction. Insufficient funding at the international and domestic levels poses a significant threat to future progress and there is growing emphasis on the need for enhanced domestic resource mobilization. The Private Sector Malaria Prevention (PSMP) project was a 3-year intervention to catalyse private sector investment in malaria prevention in Ghana.

**Methods:**

To assess value for money of the intervention, non-donor expenditure in the 5 years post-project catalysed by the initial donor investment was predicted. Non-donor expenditure catalysed by this investment included: workplace partner costs of malaria prevention activities; household costs in purchasing LLINs from retail outlets; domestic resource mobilization (public sector financing and private investors). Annual ratios of projected non-donor expenditure to annualized donor costs were calculated for the 5 years post-project. Alternative scenarios were constructed to explore uncertainty around future consequences of the intervention.

**Results:**

The total donor financial cost of the 3-year PSMP project was USD 4,418,996. The average annual economic donor cost per LLIN distributed through retail sector and workplace partners was USD 21.17 and USD 7.55, respectively. Taking a 5-year post-project time horizon, the annualized donor investment costs were USD 735,805. In the best-case scenario, each USD of annualized donor investment led to USD 4.82 in annual projected non-donor expenditure by the fifth-year post-project. With increasingly conservative assumptions around the project consequences, this ratio decreased to 3.58, 2.16, 1.07 and 0.93 in the “very good”, “good”, “poor” and “worst” case scenarios, respectively. This suggests that in all but the worst-case scenario, donor investment would be exceeded by the non-donor expenditure it catalysed.

**Conclusions:**

The unit cost per net delivered was high, reflecting considerable initial investment costs and relatively low volumes of LLINs sold during the short duration of the project. However, taking a longer time horizon and broader perspective on the consequences of this complex catalytic intervention suggests that considerable domestic resources for malaria control could be mobilized, exceeding the value of the initial donor investment.

**Supplementary Information:**

The online version contains supplementary material available at 10.1186/s12936-022-04218-2.

## Background

An estimated 1.5 billion malaria cases and 7.6 million malaria deaths have been averted globally since 2000 due to concerted global investment and control efforts [1]. Nevertheless, malaria remains one of the leading causes of morbidity and mortality worldwide and there are signs that the rate of decline has slowed since 2015 [1]. In 2017, the World Health Organization (WHO) warned that the fight against malaria had reached a crossroads [2]. Insufficient funding at the international and domestic levels poses a significant threat to future progress. In 2019, an estimated USD 3.0 billion was invested in malaria control and elimination efforts globally by governments of malaria endemic countries and international partners. Although this is an increase on the USD 2.7 billion that was invested in 2018, it still falls short of the USD 5.6 billion estimated to be required globally to stay on track towards the Global Technical Strategy milestones to reduce malaria incidence and mortality rates by at least 90% compared to 2015 levels [1, 3, 4].

One of the guiding principles of the WHO and the Roll Back Malaria (RBM) Partnership”High burden to high impact” (HBHI) response is support for enhanced domestic (and international) resource mobilization [5]. The aim is to close the funding gap in light of uncertain donor funding for malaria control [6] and the general trends towards increasing the share of domestic funding for health, particularly in countries transitioning from low- to middle-income status [7–10].

Long-lasting insecticidal nets (LLINs) contributed an estimated 68% of the 663 million clinical cases of malaria averted between 2000 and 2015 [11]. This success has brought with it a cost. It is now difficult for a household to buy an LLIN when it needs one, due in large part to donor programmes that created a dependency on free nets and reduced incentives for the retail sector to invest in LLINs. In light of uncertain future donor funding, there is a need to help restart retail sector supply chains for LLINs as one element of a broader strategy to create sustainable financing for malaria control.

Ghana has made considerable economic progress since the first free insecticide-treated net (ITN) distributions in the country in 2002, with a twofold increase in real GDP per capita from USD 986 in 2002 to USD 1884 in 2019 (constant 2010 USD) [12], moving to lower-middle income country status in 2011. Since 2003, Ghana has received a succession of Global Fund for AIDS, Tuberculosis and Malaria (Global Fund) grants with disbursements of USD 506 million for malaria [13], as well as financial support from other donors, including the UK Department for International Development (DFID, now the Foreign, Commonwealth and Development Office, FCDO), the United States President’s Malaria Initiative (PMI), United Nations Children’s Fund (UNICEF) and World Bank [1]. However, as Ghana’s economy continues to transition towards middle income, this level of external donor support is unlikely to be sustained. The government of Ghana’s long-term vision for a “Ghana Beyond Aid” seeks to empower the private sector to stimulate further growth [14]. The Private Sector Malaria Prevention (PSMP) project was a 3-year project to catalyse the private sector through three interconnected project components in order to increase resources for malaria and support the development of an LLIN market.

While private sector markets may be more sustainable in the longer term, they are argued to need short term stimulus requiring investment of public sector resources. A key question is the value for money (VFM) of such investments—which need to be considered not just over their lifetime, but also in terms of their projected future benefits. Standard economic evaluation frameworks used to explore VFM of other LLIN interventions have measured cost per LLIN delivered over the course of the project [15]. For example, economic evaluations of early social marketing projects in Malawi [16] and Tanzania [17], the Tanzanian National Voucher Scheme (TNVS) which offered women attending antenatal care a voucher to use in the retail sector [18], and a more recent evaluation of a workplace LLIN distribution programme in Zambia [19] all concluded that these strategies were “cost-effective” with economic cost to the provider per net distributed ranging from 2018 USD 5.71 to 9.46 [20]. However, this would be inappropriate for the PSMP intervention where the main focus was on catalysing expenditure by other stakeholders and developing the market in the medium to long term after the project finished.

There have been a number of investment case studies which use modelling to estimate the future benefits of increased malaria spending (or conversely the risks of declining malaria funding) in economic terms [21–24]. However, the authors are not aware of any economic evaluations of interventions to stimulate a sustainable retail sector for LLINs in the modern context of free campaign distribution, or to mobilize domestic resources for malaria prevention.

This analysis takes a novel approach in evaluating the VFM of the PSMP project by estimating the value of catalysed expenditure in comparison to the initial donor investment.

## Methods

### Intervention setting

In Ghana, household ownership of at least one ITN has steadily increased from 19% in 2006 to 74% in 2019 through the use of complementary channels: continuous distribution through antenatal clinics (ANC), child welfare clinics (CWC) and school-based distribution, and mass distribution campaigns. Fewer than 2% of ITNs in households in 2019 were from a retail outlet [25]. During the 2018 mass campaign, many individuals in urban settings refused to take the nets when they were offered, suggesting dissatisfaction with the net types offered [26].

The majority of PSMP activities focused in the urban areas of the Ashanti, Central, Greater Accra, and Western/Western North (previously Western region, prior to 2019 administrative restructure) regions of Ghana. These were chosen based on the higher presence of target customers for retail sector LLINs, that is, middle-class families with sufficient purchasing power to buy their own LLINs of different designs to the free nets offered through mass campaigns [27]. The long-term vision for the project was that reducing public sector investment in free LLINs to upper-income urban households would improve equity in ensuring resources for higher-risk, lower-income and rural households.

### Intervention description

The PSMP project was implemented between July 2016 and June 2019 and involved four areas of activity: supporting the retail sector; supporting workplace partnerships for malaria prevention; advocacy and resource mobilization; and central management and co-ordination (Table 1).

**Table 1:** Timeline for main PSMP activities by project component

Activity	Year 1 July 2016–June 2017				Year 2 July 2017–June 2018				Year 3 July 2018–June 2019			
	Q1	Q2	Q3	Q4	Q1	Q2	Q3	Q4	Q1	Q2	Q3	Q4
Inception phase												
Project design	X											
Project set-up	X											
Supporting the Retail Sector												
Engagement with LLIN manufacturers & distributors		X	X	X	X	X	X	X	X	X	X	X
Market analysis		X	X	X	X	X	X					
Public Private Partnership Stakeholder Workshop		X										
Promotion of student-sized LLINs through retail sector		X			X				X			
LLIN seed stock programme for distributors									X			X
Generic marketing design & implementation										X	X	X
Supporting Workplace Partnerships												
Malaria Safe (MS) initiative launch			X									
Subsidy system & technical support for LLIN purchases through MS partners				X	X	X	X	X	X	X	X	X
MS seminars on malaria prevention for (i) senior staff; (ii) employees				X	X	X	X	X	X	X	X	X
Peer educator training for MS partner employees				X	X	X	X	X	X	X	X	X
Web-based monitoring tool (Dashboard) developed for MS partners							X	X				
Advocacy & Resource Mobilization												
Advocacy Strategy Development Workshop		X										
Business networking and engagement events				X	X	X						
Malaria Safe Award Ceremony						X						
West Africa Regional Malaria Safe Award Ceremony								X				
World Malaria Day activities				X				X				X
Development & production of advocacy materials			X	X	X	X			X	X		
Media activities				X	X	X	X	X	X	X	X	X
PSMP Advocacy Advisory Council meetings					X	X	X	X	X	X	X	X
Support NMCP in domestic resource mobilization, including Ghana Malaria Foundation							X	X	X	X	X	X
Management & Coordination	X	X	X	X	X	X	X	X	X	X	X	X

#### Supporting the retail sector

These activities aimed to develop a sustainable retail market for LLINs in Ghana. In the absence of local LLIN manufacturers, engagement with international LLIN manufacturers resulted in Memorandums of Understanding being signed with three in-country distributors of three international LLIN manufacturers.

Major activities under this component were thorough baseline and endline market analyses to provide the retail sector with reliable and current market information, and a human-centred design study to understand consumer design preferences and willingness-to-pay for non-standard LLINs, with the aim of stimulating private sector involvement and investment. Details of the methods and findings of these studies are provided elsewhere [27–30].

PSMP facilitated the business planning and financed LLIN seed stock for two partner distributors (worth around USD 75,000 per distributor). This was a one-time start-up grant for LLIN seed stock procurement to stimulate a steady supply chain for institutional and retail sales of WHO-approved LLINs, and to reduce distributor cash flow risks associated with retail distribution. PSMP worked with an advertising agency in Ghana to develop the comprehensive generic LLIN demand creation “NetLife” campaign to support retail sales, including print, radio, TV and social media advertising. The NetLife campaign was officially launched in March 2019, complemented by mini market activations in the three regional capitals.

#### Supporting workplace partnerships for malaria prevention

PSMP engaged with registered companies and agricultural co-operatives to promote enrolment in the Malaria Safe workplace initiative, a formalized system of malaria prevention support. As part of Malaria Safe, PSMP conducted the following activities:

- Facilitated procurement of LLINs through partner distributors for distribution to employees, co-operative members or adopted communities. Subsidies of 20% for private companies (excluding those in the oil, extractive industries and financial sectors) and 40% for agricultural co-operatives were available for the first year of their participation. Costs of distribution to individual recipients were paid by the workplace partner, however technical assistance for LLIN distribution was provided by PSMP where needed.
- Developed and produced factsheets and briefs on the health and business benefits of malaria prevention.
- Facilitated seminars for senior staff and management, and employees to stimulate the interest of companies to invest in malaria prevention activities, and specifically LLINs.
- Trained workplace malaria peer educators to serve as malaria champions within companies to carry out malaria prevention education, primarily in the use and care of LLINs.
- Developed, introduced and provided technical support on a web-based Dashboard for companies to monitor malaria data, including LLIN purchases and absenteeism numbers.

By the end of PSMP, 50 workplace partners had signed up to the Malaria Safe workplace initiative.

#### Advocacy and resource mobilization

PSMP worked with existing malaria advocacy stakeholders to develop high-level advocacy activities and events. An Advocacy Advisory Council was developed, consisting of high-level media, public relations, private sector, government and non-governmental organization leaders. Advocacy activities included engagement with companies and agricultural co-operatives through promoting the Malaria Safe workplace initiative in their national or regional association meetings; raising the media profile of private sector involvement in malaria prevention; and recognition of Malaria Safe partners through high profile award ceremonies.

PSMP also worked closely with the national malaria control programme (NMCP) supporting broader resource mobilization goals, including increased domestic resources through the government and private sector. As such, PSMP supported the RBM Partnership and African Leaders Malaria Alliance (ALMA) efforts to revitalize the Ghana Malaria Foundation (GMF), which aims to raise resources for malaria interventions, and the development of an updated Resource Mobilization Strategy for Malaria Control and Elimination (2019–2023). The Resource Mobilization Strategy was validated in May 2019 and includes objectives on the market development approach for malaria commodities.

#### Central management and co-ordination

Members of the core project management team spent time on cross-cutting project or financial management activities necessary for coordination of the project as a whole.

### Framework for costing analysis

**Fig. 1:**
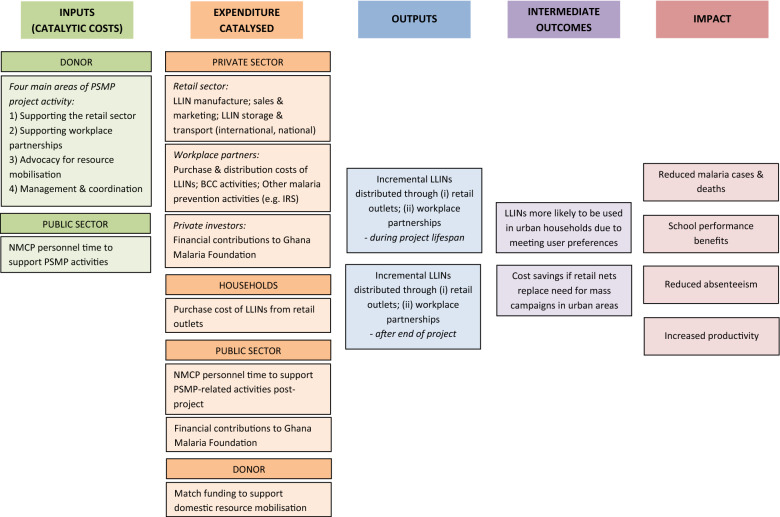
Cost-consequences framework for the Private Sector Malaria Prevention project. This provides the analytical framework for the consequences included in the catalysed expenditure analysis. Intermediate outcome and impact measures were not included

The PSMP project was a catalytic intervention to invigorate private sector investment in malaria prevention. Therefore, although the project’s costs were incurred over 3 years, relatively few of the benefits were realized within this same time-period; the main benefits are expected to continue and build over a longer timescale. A cost-consequences framework was used to map out the catalytic intervention costs to different stakeholders and the projected consequences of the intervention (Fig. 1).

In brief, catalytic costs of the intervention are presented from the donor perspective. Costs to other stakeholders are considered as consequences of this catalytic investment and include: workplace partner costs of malaria prevention activities; household costs in purchasing LLINs from retail outlets; domestic resource mobilization in terms of increased public sector financing as well as financial contributions for malaria control by private investors. The time horizon for projecting catalysed expenditure is 5 years following the end of the PSMP project, balancing sufficient time for the benefits of the intervention to be realized with the increasing uncertainty of assumptions around such benefits too far into the future. Some of these cost and consequences data could be collected empirically, whilst for others it was not possible, either due to limited funding for the evaluation or because the consequences were too far in the future. Where appropriate, estimates were taken from the published literature or programme documents and alternative scenarios constructed to explore uncertainty around future consequences of the intervention.

Final health and economic outcome and impact consequences further downstream of the initial catalytic investment are not included in this analysis which focuses on the intermediate consequences that could feasibly be achieved within 5 years of project end. For example, cost savings due to reduced malaria morbidity and mortality that result from increased LLIN ownership and use, or the economic benefits arising from reduced work and school absenteeism and increased productivity are not included (Fig. 1).

### Financial and economic costs to the donor

A top-down approach was used to identify all resources required to deliver the PSMP project interventions from the donor perspective [31]. Financial costs were obtained retrospectively from the project financial reports and accounts in Ghanaian Cedi (GHS) or United States Dollars (USD), for 3 years of intervention implementation (July 2016–June 2019).

Time spent by the Ghana- and US-based team on the four areas of project activity were estimated by individual team members each project quarter and salaries allocated proportionally. Shared costs such as office equipment, office rent, office furniture and utilities were classified as overheads and allocated across the four areas of activity proportionally to personnel time, separately for the Ghana and US teams. In Ghana, office space and capital equipment, including vehicles was shared with another project and 50% was charged to the PSMP project before allocating to specific activities.

In-depth interviews (IDIs) were conducted in July–August 2019 with stakeholders involved in PSMP-supported activities from the public sector (including members of the NMCP) and those involved in malaria advocacy (n = 3) to capture any financial or economic resources provided to PSMP-supported activities from the public sector perspective and the time commitment involved for participation in membership of the Advocacy Advisory Council, respectively.

### Value of private and public expenditure catalysed by donor investment

Financial costs incurred by workplace partners were obtained from the PSMP Dashboard, a database used by workplace partners to record expenditure on LLINs, LLIN distribution, behaviour change communication (BCC) and other malaria prevention activities. To validate the Dashboard data, estimate time spent on PSMP-related activities and gather perspectives on the sustainability of malaria prevention activities post-PSMP, IDIs were conducted in July–August 2019 with a sub-sample of these institutional partners (31 interviewees from 16 companies).

Costs to retail sector stakeholders (manufacturers, distributors, retail outlets) were not included in the analysis as it was assumed that their costs were fully recovered through profit margins applied along the supply chain. However, two PSMP partner distributors were interviewed to gather their perceptions on the sustainability of a private market for LLINs in Ghana.

By the final year of the PSMP project, LLINs with add-on features (such as zippers for easy entry, pockets, easy hanging mechanisms) had replaced standard LLINs as the main design purchased through retail outlets. It was assumed that this change in consumer demand would continue for the 5 years post-project with an average retail price of USD 7.48 (based on results of the human centred design study on LLIN preferences [28] and willingness to pay amongst middle-class households in the project area [27]). To model the financial contributions by households via LLIN purchase, assumptions about the number of households buying LLINs were made drawing on PSMP monitoring data on retail sales, and willingness to pay probabilities and least poor population estimates from the discrete choice experiment by Alonso et al. [27].

Estimates for domestic resource mobilization 5 years post-project were informed by the plans and targets described in the Resource Mobilization Strategy for National Malaria Control and Elimination (2019–23) [32]. The strategy rates potential resource mobilization measures for applicability and feasibility; to project the catalysing effect of PSMP, the costs of the measures rated in the strategy as “most feasible” for the public and private sector were estimated. Over the timeframe for this analysis, the most feasible source of increased public spending on malaria was rated to be activating the 0.5% of District Assembly Common Funds (DACF) ear-marked for local-level malaria control activities (0.5% of the 2018 DACF budget nationally came to USD 1,757,661) [32]. The Resource Mobilization Strategy assessed the most feasible potential sources of private investment in the near-term to be match funding programmes where every USD 1 of private donation would be matched by USD 1 from international donors or foundations [33], corporate fundraising (e.g. a recent telethon led by Ecobank Ghana), and investments by philanthropists and diaspora [32]. These various private investments would be coordinated by the Ghana Malaria Foundation using a public–private partnership model, whereby funds will be used in alignment with the national malaria strategic plan via a Technical Oversight Committee chaired by the NMCP.

### Analysis

Costs were collected in the currency of expenditure. Costs in GHS were adjusted for inflation to 2019 GHS using consumer price indices available from the International Monetary Fund [34] and then converted to 2019 USD using the official average annual exchange rate for 2019 (1 USD equivalent to GHS 5.03 [35]) [31]. Costs in USD were adjusted for inflation and are presented as 2019 USD using consumer price indices available from the International Monetary Fund [34]. Capital goods with an expected lifespan of more than 1 year were annualized using a discount rate of 3% [36]. A useful lifespan of 8 years was used for vehicles and office equipment, and 10 years for furniture [37].

### Cost per LLIN distributed during the project

The number of LLINs delivered through retail and workplace partners during the 3 years of the project was reported by PSMP annually, based on data collected on a routine basis from the project’s partners:

- LLIN distributors reported on the number of LLINs sold to retail outlets
- LLIN distributors and institutional partners reported on the number of LLINs distributed through Malaria Safe workplace initiatives; numbers from these two sources were triangulated to avoid double-counting

Estimates of numbers of LLINs sold to retail outlets and distributed by workplace partners in the year prior to PSMP were also obtained. It was assumed that without the catalytic activities of PSMP, the annual volumes of LLINs sold through retail outlets would have remained constant, so pre-PSMP volumes were subtracted from annual intervention-period volumes to calculate the *incremental* number of LLINs delivered through retail outlets due to the PSMP project. For the workplace partners, information on pre-project LLIN distributions was obtained via baseline questionnaires with the partners: 47 of 50 workplace partners completed the questionnaire, of these only 4/47 reported that they had previously purchased nets. As the majority of workplace partners were distributing LLINs for the first time, it was assumed that all LLINs purchased through PSMP were due to the project activities.

Total financial costs to the donor of supporting the retail sector or workplace partners were divided by the incremental number of LLINs distributed to retail outlets or workplace partners during the 3 years of project implementation to estimate the donor’s financial cost per LLIN delivered through each of these respective channels.

To estimate average annual economic cost over the effective lifespan of the LLINs delivered through the retail sector or workplace partners, project costs supporting these activities were annualized across the average LLIN retention time in Ghana of 1.78 years [38] using a discount rate of 3% [37]. This reflects that the donor-supported costs are investments expected to last as long as the LLINs.

### Predicted expenditure catalysed by the donor investment in the 5 years post-project

The activities and outcomes of the project were broader than those reflected through cost per net distributed during the lifespan of the project, reflecting its goal to catalyse private sector investment in malaria control. The intermediate consequences of the donor investment in PSMP-supported activities were brought together by calculating annual ratios of non-donor expenditure to donor expenditure for the 5 years after project end.

To calculate annual donor investment costs, the donor-funded costs of the PMSP intervention were treated as investment (capital) costs and annualized using a discount factor of 3% across the lifespan of the intervention, adjusted for the year in which the cost was incurred. For example, costs incurred in year one of the project were divided by 7.02 (8 years discounted at 3%) reflecting that the effects of these activities would last for three years of project implementation plus 5 years post-project. Costs incurred in years two and three of the project were divided by 6.23 and 5.42, respectively (reflecting discounted lifespan of these activities of 7 and 6 years, respectively).

Five scenarios were created for the consequences of the PSMP intervention in terms of annual recurrent expenditure by all stakeholders (donor, public, private and households) for the 5 years after project implementation ended. The assumptions behind the five scenarios ranged from optimistic but feasible increases in non-donor expenditure in the best case to no change in the status quo in the worst case, as summarized in Table 2. The full list of annual recurrent costs to different stakeholders for the 5 years post-project are provided in Additional File 1, along with the estimated values for each scenario, and the data sources and assumptions.

**Table 2:** Overview of scenarios and assumptions for projections of expenditure in years one to five post-project

Scenario	Description of assumptions
Best case	- Minor donor support to retail sector, workplace partnerships & GMF in Y1-Y2 post-project; - Support moves to NMCP or self-financing from GMF funds in Y3-Y5; - Optimistic but feasible growth each year in household purchase of LLINs with add-on features from retail sources; - Increase of 20% each year in workplace partner contributions; - Public investments to the GMF from % of DACF (0.5% in Y1-Y3, 0.75% in Y4, 1.0% in Y5); - Match funding initiative in Y3, with private investments matched 1:1 by donor funding; - Private investments to the GMF start in Y3 and increase by 10% each year (corporate & individual fundraising)
Very good case	- Minor donor support to retail sector, workplace partnerships & GMF in Y1-Y2 post-project; - Support moves to NMCP or self-financing from GMF funds in Y3-Y5; - Conservative growth each year in household purchase of LLINs with add-on features from retail sources; - Increase of 15% each year in workplace partner contributions; - Public investments to the GMF from % of DACF (0.5% in Y1-Y5); - Match funding initiative in Y3, with private investments matched 1:1 by donor funding - Private investments to the GMF start in Y3 and increase by 5% each year (corporate & individual fundraising)
Good case	- Minor donor support to GMF in Y1-Y2 post-project; self-financing from GMF funds in Y3-Y5; - No donor support for retail sector or workplace partnerships; - Minimal growth each year in household purchase of LLINs with add-on features from retail sources; - Increase of 10% each year in workplace partner contributions; - Public investments to the GMF from % of DACF (0% in Y1-Y2, 0.25% in Y3, 0.5% in Y4-Y5); - Match funding initiative in Y3, with private investments matched 1:1 by donor funding - Private investments to the GMF start in Y3 and increase by 2.5% each year (corporate & individual fundraising)
Poor case	- Minor donor support to GMF in Y1-Y2 post-project; self-financing from GMF funds in Y3-Y5; - No donor support for retail sector or workplace partnerships; - Household purchase of LLINs with add-on features from retail sources remains at same level as final year of project (no growth); - Workplace partner contributions remain same level as final year of project (no growth); - No public investments to the GMF from % of DACF; - Match funding initiative in Y3, with private investments matched 1:1 by donor funding - Private investments to the GMF start in Y3, remain constant in Y4-Y5 (corporate & individual fundraising)
Worst case	- No donor support for retail sector, workplace partnerships or GMF; - Household purchase of LLINs with add-on features from retail sources remains at same level as final year of project (no growth); - Workplace partner contributions remain same level as final year of project (no growth); - No public investments to the GMF from % of DACF; - No private investments to the GMF (corporate & individual fundraising) or match funding

*DACF* district assembly common fund, *GMF* Ghana Malaria Foundation, *LLIN* long-lasting insecticidal net, *NMCP* national malaria control programme, *Y* year (number of years after end of project)

The catalysed expenditure analysis was drawn together to produce an annual ratio of non-donor expenditure to donor expenditure using the following simple formula for each year post-project:

Non-donor: donor expenditure ratio = (annual recurrent expenditure by households + public sector + private investors)/(annualized donor investment cost + annual recurrent donor expenditure).

A ratio greater than one indicates that the PSMP project catalysed greater public and private investment in malaria prevention than the donor investment. Conversely, a ratio less than one indicates that donor investment costs exceeded catalysed expenditure.

### Sensitivity analysis

To investigate the effect of assumptions made on the annual non-donor to donor expenditure ratios, each was varied in one-way sensitivity analyses. The base case discount rate of 3% used to annualize donor investment costs was varied to 0% and 5% [31]. The proportion of households willing to purchase an LLIN with add-on features, and price per LLIN with add-on features that these households were willing to pay were varied by ± 25% of the base case parameter value [27].

## Results

### Costs of intervention

The total financial cost of the PSMP project over the 3 years of implementation (July 2016–June 2019) was USD 4,418,996. Supporting the retail sector accounted for 37% of these costs, management and co-ordination 26%, advocacy and resource mobilization 20% and supporting workplace partnerships 17%.

Personnel costs (PSMP staff salaries and per diems for field travel) represented the greatest proportion of total costs for all four components, although they were highest for the workplace partners component and management and coordination component at 59% and 55%, respectively (Table 3). Charges for the agencies contracted to run the two high level Malaria Safe award ceremonies that took place to recognize private sector contributions to malaria control comprised 28% of the costs of the advocacy and resource mobilization component; agency charges for the market analysis comprised 12% of the supporting the retail sector component. Costs of promotional materials and events were 10% and 21% of these components, respectively. Overheads (Ghana and US project office running costs) contributed 42% of the management and coordination costs and around 15% of the other three project components.

**Table 3:** Overview of financial donor costs of the PSMP project, by component and year (2019 USD)

	Year 1 (July 16–June 17)		Year 2 (July 17–June 18)		Year 3 (July 18–June 19)		Total	
	2019 USD	%^1^	2019 USD	%^1^	2019 USD	%^1^	2019 USD	%^1^
Supporting the retail sector								
Personnel	179,742	52	189,007	45	172,081	20	540,831	34
Transport	19,145	6	36,878	9	55,924	7	111,947	7
Agency charges	36,339	11	82,701	19	73,622	9	192,662	12
Promotional materials & events	24,725	7	3,962	1	303,228	36	331,914	21
LLINs	0	0	0	0	150,442	18	150,442	9
Overheads	78,616	23	97,958	23	76,548	9	253,122	16
Other	4,791	1	13,696	3	11,610	1	30,097	2
SUB*-*TOTAL	*343,357*		*424,201*		*843,456*		*1,611,014*	
Supporting workplace partnerships								
Personnel	144,967	65	190,288	52	117,551	68	452,807	59
Transport	31,615	14	47,178	13	24,429	14	103,223	14
LLINs	0	0	82,228	22	214	0	82,442	11
Overheads	36,636	16	43,694	12	26,084	15	106,413	14
Other	10,478	5	2,399	1	4,132	2	17,008	2
SUB*-*TOTAL	*223,695*		*365,788*		*172,410*	*9*	*761,894*	
Advocacy & resource mobilization								
Personnel	57,287	41	134,527	36	124,439	34	316,253	36
Transport	5,364	4	23,774	6	29,392	8	58,530	7
Agency charges	30,639	22	75,893	20	137,899	37	244,430	28
Promotional materials & events	17,922	13	53,688	14	14,161	4	85,770	10
Overheads	24,407	18	62,247	17	46,973	13	133,626	15
Other	2,830	2	23,561	6	16,778	5	43,169	5
SUB*-*TOTAL	*138,448*		*373,690*	*25*	*369,641*		*881,779*	
Management & co-ordination								
Personnel	198,192	62	199,201	55	247,796	51	645,188	55
Transport	11,436	4	8,632	2	5,703	1	25,771	2
Overheads	109,782	34	153,322	42	230,247	48	493,351	42
*SUB-TOTAL*	*319,410*		*361,155*		*483,745*		*1,164,310*	
GRAND TOTAL	1,024,910		1,524,834		1,869,252		4,418,996	

### Financial and economic cost per LLIN distributed during project and cost drivers

Based on distributors’ routine reports a total of 47,536 LLINs were sold to retail outlets by PSMP partner distributors over the 3-year project implementation period. This compares to distributor estimates of approximately 1000 LLINs per year prior to PSMP (Table 4). Assuming that without PSMP, annual retail sales would have remained constant, the total incremental number of LLINs sold through retail outlets in the project regions was 44,536, or an average of 14,845 per year. The total financial costs of the activities supporting the retail sector were USD 1,611,014 (Table 3). This gives a total financial cost of the catalytic donor investment of USD 36.17 per LLIN sold through retail outlets. Annualizing the financial donor costs across a 1.78-year useful lifespan for the LLINs distributed gives an economic donor cost per year of LLIN protection of USD 21.17 per LLIN sold through retail outlets. Personnel, promotional materials and events (NetLife campaign), overheads, and agency charges (market analysis) made up a large proportion of unit costs (Table 3).

**Table 4:** LLINs distributed through PSMP-supported private distribution channels

Distribution channel	Pre-PSMP	Year 1 (July 2016–June 2017)	Year 2 (July 2017–June 2018)	Year 3 (July 2018 – June 2019)	Total (Years 1–3)
Number of LLINs sold by distributors to retail outlets [Source: Distributor reports]	1,000^1^	1513	15,883	30,140	47,536
Number of LLINs distributed through workplace partners^2^ [Source: PSMP monitoring database]	8,904^3^	15,740	24,764	55,064	95,568
*Private companies–subsidy ineligible*		*0*	*18,764*	*11,114*	*29,878*
*Private companies–subsidy eligible*		*12,480*	*1,000*	*1,300*	*14,780*
*Agri-business and agricultural co-operatives*		*3260*	*5,000*	*42,650*	*50,910*

^1^Annual sales through retail outlets

^2^Private companies were eligible for subsidies of up to 20% and agricultural co-operatives for up to 40% for the first year of their participation; companies in the oil, extractive industries and financial sectors were not eligible for a subsidy. Italic values indicate that numbers of nets distributed by each category of workplace partner are provided in the final three rows of the table

^3^LLINs purchased by workplace partners for their employees and/or surrounding communities in 2016. This information was obtained via baseline questionnaires with workplace partners: 47 of 53 workplace partners completed the questionnaire, of these 4/47 reported that they had previously purchased nets

Based on PSMP financial records and partner reports, a total of 95,568 LLINs were purchased by institutions through Malaria Safe workplace partners for their employees, co-operative members, families, or adopted communities during the 3 years of PSMP implementation. Numbers increased each year from 15,740 in year one, to 24,764 in year two and 55,064 in year three (Table 4). Data provided by the workplace partners upon enrolment in the project estimated that 8,904 LLINs were distributed in 2016 prior to joining PSMP. These were distributed by four of the workplace partners (43 had not previously distributed LLINs; 3 did not provide data). As the majority of workplace partners had never distributed LLINs, all those purchased through PSMP were considered to be attributable to the project so that the total incremental number of LLINs distributed through workplace partners across the 3 years of implementation was 95,568.

The total financial cost of the project activities supporting workplace partners (USD 761,894, Table 3) plus advocacy activities targeting engagement of such workplace partners (USD 475,802, Additional File 2) was USD 1,237,696. This includes USD 82,442 in LLIN subsidies that the project provided to partner companies and agricultural co-operatives (via agricultural businesses). The incremental number of LLINs distributed through PSMP Malaria Safe workplace initiative partners over the same time period was 95,568. This gives a total financial cost of the catalytic donor investment of USD 12.91 per LLIN distributed through workplace partners and an economic donor cost per year of LLIN protection of USD 7.55 per LLIN distributed. The main driver of project-supported unit costs was personnel, followed by promotional materials and events (award ceremonies and other high-level advocacy to engage stakeholders) (Table 3).

LLIN purchase costs contributed by the project in subsidies were an average of USD 0.86 per LLIN. The workplace partners themselves paid an average additional USD 4.28 per LLIN for the remaining bulk purchase costs of LLINs and their distribution to recipients (specifications and unit costs of LLINs purchased by Malaria Safe partners varied). Thus, approximately 83% of the LLIN purchase cost was paid by the companies themselves and 17% by the project in subsidies (Additional File 3).

### Predicted expenditure catalysed by donor investment in the 5 years post-project

Taking a 5-year post-project time horizon, the annualized donor investment costs of the PSMP project were USD 735,805 (see Additional file 4 for details). In the best case scenario (Table 5), it is assumed that there would be annual recurrent costs to the donor of USD 53,388 in the first 2 years post-project to provide support to the retail sector, workplace partners and GMF secretariat to facilitate continued stakeholder engagement and technical support as required; in years three, four and five post-project these costs would transfer to the NMCP and GMF. The greatest annual contribution by the public sector will be the malaria allocation from the DACF. In years one, two and three post-project it is assumed that the current commitment of 0.5% is mobilized, amounting to approximately USD 240,000 per year; in years four and five it is assumed that continued advocacy efforts are successful in increasing this allocation to 0.75% (approximately USD 363,000) and 1% (USD 484,000), respectively.

**Table 5:** Projected annual recurrent expenditure for all stakeholders and annualized donor investment cost used to calculate ratio of non-donor to donor expenditure, based on assumptions for the best case scenario of consequences 5 years post-intervention

	Annual expenditure for each year post-project (2019 USD)				
Activity and stakeholder	Year 1	Year 2	Year 3	Year 4	Year 5
Supporting the retail sector					
Donor support to stakeholder engagement & workshops^1^	11,610	11,610	0	0	0
NMCP support to stakeholder engagement & workshops^1^	0	0	5,805	5,805	5,805
LLIN purchase costs to households^2^	450,894	901,789	1,803,578	1,803,578	1,803,578
Supporting workplace partnerships					
Donor support to stakeholder engagement & workshops^1^	16,778	16,778	0	0	0
NMCP support to stakeholder engagement & workshops^1^	0	0	8,389	8,389	8,389
Annual LLIN costs to workplace partners^3^	304,015	364,818	437,782	525,338	630,406
Annual BCC activities (seminars etc.)^3^	52,152	62,582	75,098	90,118	108,141
Other malaria prevention activities^3^	181,155	217,386	260,863	313,035	375,643
Promotional materials	13,185	13,185	13,185	13,185	13,185
Advocacy for resource mobilization					
District Assembly Common Funds for malaria^4^	241,807	241,807	241,807	362,711	483,615
Donor support to Ghana Malaria Foundation (GMF) Secretariat^5^	25,000	25,000	0	0	0
Donor support for GMF match funding^6^	0	0	750,000	0	0
Private investments & fundraising for GMF^7^	0	0	850,000	110,000	121,000
Total recurrent donor expenditure [A]	53,388	53,388	750,000	0	0
Total recurrent public sector expenditure [B]	241,807	241,807	256,001	376,905	497,809
Total recurrent private sector expenditure [C]	1,001,401	1,559,760	3,440,506	2,855,254	3,051,953
Annualized donor investment cost^8^ [D]	735,805	735,805	735,805	735,805	735,805
Ratio non-donor: donor expenditure [(B + C)/(A + D)]	1.58	2.28	2.49	4.39	4.82

^1^Assumes donor support for stakeholder engagement activities continues in years 1–2 post-project at the same cost as final project year; these activities then transfer to NMCP with half the cost in years 3–5 post-project

^2^Assumes proportion of households that buy an LLIN with add-on features from a retail outlet doubles each year in years 1–3 then remains constant in years 4–5 (see Additional file 1 for further details)

^3^Assumes 20% annual increase in workplace partner costs from final project year levels

^4^Assumes DACF for malaria are allocated as currently intended (0.5% in years 1–3 post-project; increasing to 0.75% in year 4 and 1.0% in year 5)

^5^Assumes there is donor support to initially fund a project manager to coordinate day-to-day running of GMF business; once the GMF is raising substantial donations, a proportion can be used to fund this position (as recommended in the Resource Mobilization strategy [32])

^6^Assumes GMF active by year 3 post-project and advocacy efforts successful in raising USD 750,000 from match funding programme in year 3 + further USD 100,000 from corporate fundraising and private investments from diaspora, philanthropists; this fundraising continues with a 10% annual increase in years 4–5 post-project

^7^Donor contribution to match funding programme (one dollar for every dollar donated by private investors)

^8^See Additional file 4 for details of annualized donor investment costs

In the best case scenario, private sector contributions are substantial, increasing from USD 1,001,401 in the first-year post-project to USD 3,051,953 in the fifth-year post-project. This relies on optimistic but feasible assumptions on the set-up and success of the GMF, and continued engagement of workplace partners and retail sector stakeholders (manufacturers, distributors and customers) as a result of the annual donor- and public sector-funded supportive activities. For example, it was assumed that the workplace partner activities would increase by 20% each year from the final project year levels. Supply and demand for LLINs with add-on features would grow year-on-year, from an estimated 5% of households willing to buy a differentiated LLIN in the first year post project to 20% in the fifth year.

**Fig. 2:**
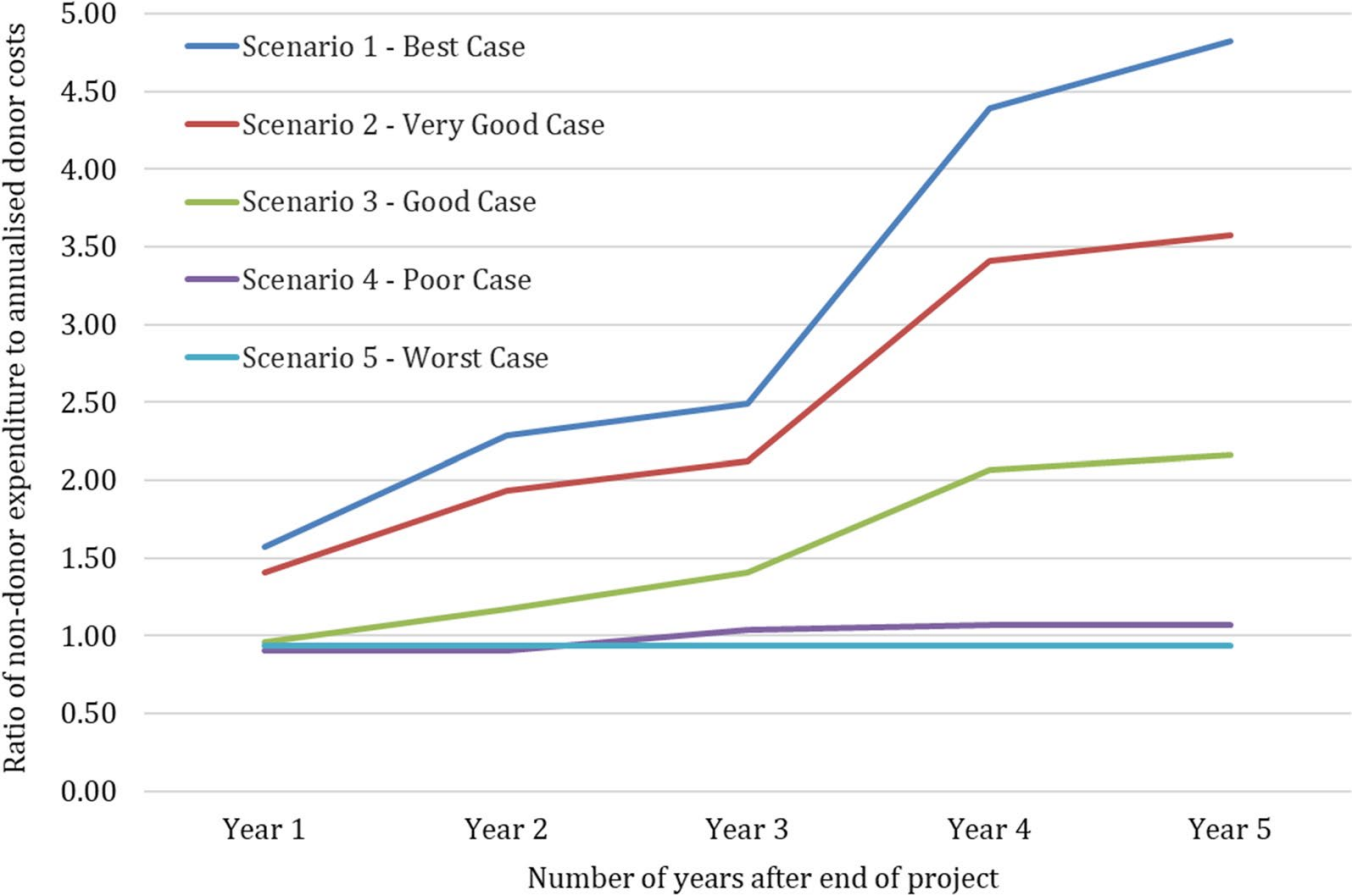
Ratio of annual non-donor to donor expenditure for 5 years after project end. Projected ratios presented for five scenarios ranging from best case to worst case according to assumptions for non-donor contributions (see Table 5 and Additional File 1 for details of scenario assumptions)

Given these assumptions, the ratio of annual non-donor to donor expenditure is greater than one for all 5 years post-project, increasing from 1.58 in year one to 4.82 in year five (Table 5, Fig. 2). Essentially, this means that for every dollar of donor investment in PSMP-related activities, by the fifth-year post-project there is a return of USD 4.82 in public- and private-sector spending on malaria (based on the projected expenditure included in this analysis).

In the scenario analysis, the assumptions behind the projections of donor, public sector and private expenditure were gradually made more conservative in comparison to the best case scenario, for example, by reducing the assumed percentage growth in retail sales, workplace partner initiatives and private contributions to the GMF. Nevertheless, for all scenarios except the worst case scenario the ratio of non-donor to donor expenditure remained above the "break-even" ratio of 1 from the third year post-project (Fig. 2). For the worst case scenario, which assumed that retail sales and workplace partner contributions would remain at the same levels as the final project year and the GMF would remain inactive with zero domestic resource contributions, the ratio of non-donor to donor expenditure remained below 1 (0.93 in years one to five post project).

Annual non-donor to donor expenditure ratios were sensitive to the discount rate, retail price of LLINs with add-on features and proportion of households willing to pay for an LLIN with add-on features. However, in the best case scenario, annual non-donor to donor cost ratios remained well above the “break-even” ratio of 1 from the first year post-project; the same sensitivity analyses on the good case scenario also resulted in annual non-donor to donor cost ratios greater than one for all years except the first year post-project. The annual non-donor to donor expenditure ratios for the poor case scenario were at or just above one in all years except the first and second year post-project. This shows that if the retail sector and workplace partner gains made by the final year of the PSMP project lifespan are maintained at the same level and there is some modest level of donations through the GMF, the donor investment in PSMP will still be above the break-even threshold over a 5 year time horizon and this is robust to uncertainty in certain key parameters.

## Discussion

The PSMP project was a package of interventions to catalyse private sector investment in malaria prevention in Ghana. The study compared the costs of the PSMP project, over the 3 years of its implementation, to the non-donor expenditure on malaria prevention catalysed as a result.

A traditional metric of value for money of LLIN distribution is cost per LLIN distributed. The average annual economic donor cost per LLIN distributed through workplace partners in this project was USD 7.55. This compares favourably with the limited other evidence of costs of workplace LLIN distribution, for example an intervention that distributed LLINs to cotton farmer cooperative members in Zambia found an annual provider cost per LLIN distributed of USD 5.28 [19]. The average annual economic donor cost per LLIN distributed through retail outlets during the project implementation period was USD 21.17 which is high compared to the small number of economic evaluations involving the retail sector that exist from the era before universal coverage targets for ITNs and the large donor commitments that followed. For example, annual economic donor cost per net through subsidized sales supported by social marketing was USD 6.13 (adjusted to 2019 USD) in Burkina Faso [39] and USD 4.05 in Malawi [16], and USD 9.65 per net delivered through the Tanzania Net Voucher Scheme (TNVS) which offered women attending antenatal care a voucher to use in the retail sector [18]. For comparison, a recent review by Wisniewski et al. found that the median annual economic donor cost per LLIN distributed through mass campaigns was USD 4.13 and through public sector continuous distribution channels was USD 5.00 (adjusted to 2019 USD) [40].

The higher unit donor cost found for LLINs distributed through the retail sector as a result of the PSMP project is largely due to the costs and prolonged timespan of the market analysis, which delayed the launch of many of the retail activities, and the costs associated with developing the generic demand generation campaign for a relatively modest number of LLINs distributed. However, both the market analysis and generic demand creation campaign were important investment costs that were highly valued by the partner distributors. The full benefits of these activities in terms of LLIN sales had not been realized by the end of the project; continued LLIN sales growth catalysed by the PSMP-funded activities would reduce the unit costs. Similarly, the project never intended to distribute as many nets as a mass campaign or routine channels; private sector distribution is intended to be complementary and reach a different audience. Therefore, comparison of unit costs per LLIN delivered through this project during its 3 year period of implementation with other LLIN distribution strategies fails to capture the full value for money of the intervention.

A large focus of the PSMP project was on advocacy, promoting the economic benefit through improved productivity of investing in malaria control amongst medium- and large-sized businesses in Ghana [21, 22, 41], raising the profile of businesses that engaged in malaria control as a result of their involvement in PSMP-funded activities, and working with other stakeholders to develop the national Resource Mobilization Strategy. The consequences of these advocacy activities were brought together with the retail and workplace partner activities in the catalysed expenditure analysis.

In the best case scenario, each USD of annualized donor investment led to USD 4.82 in annual non-donor expenditure by the fifth year post-project. With increasingly conservative assumptions around the project consequences, this ratio decreased to 3.58, 2.16, 1.07 and 0.93 in the “very good”, “good”, “poor” and “worst” case scenarios, respectively. This suggests that in all but the worst case scenario (which assumed that the benefits from the project do not continue—retail sales and workplace partner activities do not grow beyond the final project year level, and the GMF remains inactive), there will be some level of return on the donor investment.

There are a number of assumptions included in the scenario testing of the catalysed expenditure analysis. The aim was to balance optimism with feasibility based on stakeholder interviews and knowledge of the national and international context. For example, regarding the trajectory of retail sales of LLINs with add-on features: a number of challenges remain with establishing a viable retail market for LLINs in Ghana, including access to credit for distributors, and exchange rate fluctuations making price/margin setting difficult. Nevertheless, the distributors were optimistic that the relationships made during the PSMP project, for example with large wholesalers and their networks of retail outlets (mostly pharmacies) and the workplace partners, will continue after the project. It therefore seems reasonable to assume that there will be some degree of growth in the market for LLINs with add-on features. Distributors noticed that their initial seed stocks of LLINs with add-on features (designed by manufacturers as a result of the human-centred design results from the PSMP-funded market analysis) sold quickly, commenting that they wish they had shown greater faith in the market analysis findings and ordered more of these. Maintaining distributor and manufacturer interest in these LLINs with add-on features and encouraging their marketing in suitable target areas to promote demand could achieve the LLIN sales predicted in the catalysed expenditure analysis. However, it is likely that some external support for this will still be required.

Similar comments were made by workplace partners interviewed as part of the PSMP end-of-project evaluation: there was pride in the achievements made during their involvement in the project and a desire to continue. However, there was also recognition from some companies that they would require continued technical support. This could in theory be provided by the NMCP, however the NMCP has many competing priorities for their time which may prove a challenge.

Along with other stakeholders, PSMP supported the development and validation of the national Resource Mobilization Strategy for Malaria Control and Elimination (2019–2030), which includes engaging private institutional and individual investors to help fill the financing gaps in the National Strategic Plan as well as other innovative ways to generate finances for malaria control. The Ghana Malaria Foundation (GMF) is the mechanism to manage such private financial donations from various sources which could be accessed by the NMCP for malaria control activities. There have been challenges in finalizing the GMF, however once this system is in place and with continued advocacy work there is potential for considerable domestic resource mobilization in Ghana. The assumptions made in the catalysed expenditure analysis about levels of public and private domestic resources which could be mobilized in the 5 years post-project were largely based on recommendations made in the national Resource Mobilization Strategy and therefore on expert opinion about realistic feasibility from senior political and business leaders. For example, the malaria allocation from the district assembly common funds (public sector resources) were included along with private donations, corporate fundraising and match funding initiatives which were rated highly for being applicable and feasible [26]. Other options for domestic resources (such as small transaction charges on remittances sent from overseas, earmarked tax revenues for malaria) were not included as the national Resource Mobilization Strategy concluded that these were not currently feasible in the near- to medium-future.

In considering all of the assumptions behind the catalysed expenditure analysis and the projected expenditure catalysed by the initial donor investment, it is important to note the disruption of the Covid-19 pandemic on what could feasibly have been achieved in the early post-project period. One could argue that the “good case” is the most realistic, where non-donor expenditure in the first 2 years post-project (2020–2021) are slow but pick up in years three to five (2022–2025). In this scenario, the non-donor expenditure catalysed is still over two-fold the initial donor investment.

Making direct comparisons of the unit costs of nets distributed as a result of the PSMP intervention with those of nets distributed through other channels (such as mass campaigns or antenatal clinics) as a means of assessing the value of the investment is problematic and does not align with the goal of the project or this costing analysis. For example, even if the cost per LLIN delivered through retail outlets or workplace partners was lower than the cost per LLIN delivered through a mass campaign or routine delivery channel (which it was not), there are equity and efficiency concerns to consider. There is a long history to the debate on whether there should be a private sector for the sale of insecticide-treated nets at all given their public health value and the disproportionate burden of morbidity and mortality amongst the poorest populations who cannot afford to pay [42–44]. Under the current guidelines from the WHO, NMCPs continue to pursue universal coverage with an emphasis on free mass campaigns complemented by continuous distribution [4]. Private sector channels are important to understand because of their potential in terms of sustainability and independence from donor decisions which can abruptly disrupt supplies. Similarly, a private retail market gets those who are not using standard nets and can afford to buy LLINs with add-on features to actually use nets thus increasing overall coverage. The authors are, therefore, not suggesting that money currently being spent on efforts to achieve universal coverage with LLINs through free universal LLIN campaigns or routine distribution channels is diverted to the private sector. However, if middle-class households in urban areas purchase their own LLINs that they are more likely to use due to design preferences [28], there is the potential for costs savings to the NMCP if these populations were no longer recipients of free nets. These cost savings, along with increased domestic resource mobilization could then be used for malaria control in poorer areas with the potential for greater health impact of the NMCP as a whole. It is important to note that although there is a greater move towards tailoring malaria control interventions according to stratification of risk [5], specific policy recommendations and guidelines around strategic use of retail channels for achieving LLIN coverage in certain areas (such as wealthy urban neighbourhoods) are not yet in place; the political and operational considerations are complex and likely to take years to resolve.

In this catalysed expenditure analysis, no assumptions were made about cost savings to the public sector from retail LLIN sales or workplace distributions, likewise the consequences further downstream of the initial catalytic investment are not included in this analysis. For example, to fully understand the additional benefit of LLINs distributed through retail channels or workplace partners, one would need to know the additional number of people sleeping under a net as a result of the intervention and the vulnerability of those individuals to malaria i.e. were the private sector nets additional to those delivered through public channels, or substitutes? A comparison of LLIN ownership and use by source of net before and after the PSMP intervention would have been required to answer this question. Given the relatively low volumes of LLINs directed through the retail outlets and workplace partners, and the geographically-focused nature of the intervention, the resources required for such an evaluation could not be justified.

Attribution of health and economic impacts to any one particular intervention gets harder the further downstream the consequences are from the intervention itself. For example, understanding if there was additional net ownership and use due to the project activities is challenging in the context of multiple LLIN distribution channels; improvements in absenteeism and productivity could be due to broader workplace public health initiatives or general improvements in population health, particularly in least poor areas targeted by the intervention where standards of living and housing conditions are rising. For these reasons, the catalysed expenditure analysis was limited to a 5-year time horizon and to the consequences more easily attributable to the PSMP activities. However, by excluding the longer-term consequences, the full long-term impact of the catalytic donor investment is likely to be underestimated.

Likewise, with the exception of the assumptions made about private investment into the GMF (which will operate at the national level), the geographical scope of consequences included in the catalysed expenditure analysis are limited to the least poor districts of Ashanti, Greater Accra and Western regions (the main project area). Although these areas were selected for the project due to having the highest concentration of private businesses and greater proportions of middle-class populations than the other regions of Ghana [30], there could still be the potential to scale-up stakeholder engagement with workplace partners and retail stakeholders more broadly across Ghana and thus also scale-up the positive consequences of the catalytic investment. The LLIN distributors supported by the PSMP project were the only distributors of WHOPES-approved LLINs in Ghana at the time and so shared the advantages of PSMP support without undercutting other distributors to retail outlets not part of the project.

Although the GMF was not up and running by the end of the project, there is continued momentum driven by the changing funding landscape for malaria and Ghana’s Beyond Aid Agenda. A recent investment case analysis by Shretta et al. estimated that elimination of malaria in Ghana will cost USD 961 million between 2020 and 2029, approximately USD 133 million annually for the first 5 years, with a huge economic return at 32 times the investment; conversely, reducing investment will lead to resurgence in cases and economic losses [21]. In 2019, Ghana received around USD 36 million from the Global Fund and USD 29.5 million from other donors [1], suggesting a shortfall of approximately USD 67.5 million which will need to be met domestically; in 2019, government spending on malaria was around USD 10.8 million [1].

The international context is one of diminishing resources for malaria control [6] and it is possible that policies and guidance for programme planning may change in coming years. There is growing high-level political commitment to increase domestic financing for malaria as exemplified through the work of ALMA. Lessons learned from the PSMP project may be of value to other countries looking to catalyse non-donor expenditure for malaria control as these discussions evolve internationally.

## Conclusions

The average annual economic donor cost per net delivered through retail outlets during the project was high compared to other distribution channels. Average annual economic donor cost per net delivered through workplace programmes were comparable to other LLIN distribution channels. However, the authors argue that this metric is not suitable to capture the full value for money of a complex catalytic intervention such as PSMP.

As this catalysed expenditure analysis has shown, donor investments in the above activities have the potential to catalyse up to five-fold non-donor expenditure for malaria control for each dollar spent over a 5-year post-project time horizon. In the best case scenario of this catalysed expenditure analysis, by the fifth year post-project annual non-donor expenditure (to public sector, private investors and households) was estimated to be USD 4.82 for every USD 1 invested by the donor. Even with more conservative assumptions about non-donor spending (and some small continued donor or public sector investments to support private sector engagement), non-donor spending of USD 2.16 for every USD 1 donor investment by year five post-project was estimated. However, it is important to note that some level of facilitation between public and private stakeholders is likely to be needed to maintain gains that can be made during a 3-year project period. It would be valuable to conduct a further evaluation of the situation in Ghana to test these assumptions and provide further evidence of the sustainability of efforts to catalyse domestic mobilization of resources for malaria control which is going to be of increasing importance for Ghana and other countries given the changing malaria funding landscape.

## Supplementary Information

**Additional file 1.** Annual recurrent costs by activity and stakeholder.

**Additional file 2. **Allocation of advocacy activities between (i) supporting workplace partnerships and (ii) broader domestic resource mobilisation activities (2019 USD).

**Additional file 3: **Share of purchase costs of LLINs distributed through workplace partners over the three years of project implementation (2019 USD).

**Additional file 4. **Annualised donor investment costs.

## Data Availability

The datasets used and/or analysed during the current study are available from the corresponding author on reasonable request.
