# Supplementary material for: Using donor funding to catalyse investment in malaria prevention in Ghana: an analysis of the potential impact on public and private sector expenditure
Source: Malar J. 2022 Jun 27;21:203. doi: 10.1186/s12936-022-04218-2 (PMC9235193; doi:10.1186/s12936-022-04218-2)
Supplement: Supplementary file 1 — Additional file 1. Annual recurrent costs by activity and stakeholder. [file 12936_2022_4218_MOESM1_ESM.docx]

Additional File 1: Annual recurrent costs by activity and stakeholder

| **Parameter** | **Parameter value** | **Source** |
| --- | --- | --- |
| **Costs to households** |  |  |
| Cost per HH (WTP for one LLIN with add-on features) (+/-25%) | $7.48 | PSMP DCE paper [27] |
| Number of HHs in 32 least poor districts (Ashanti, Western, GA) | 2,620,313 | PSMP DCE paper [27] |
| % not in poverty | 95% | PSMP DCE paper [27] |
| % willing to buy LLIN with add-on features (+/-25%) | 44% | PSMP DCE paper [27] |
| No. HHs willing to buy LLIN with add-on features | 1,094,138 |  |
| No. LLINs sold through retail outlets in year 3 of project | 30,140 | PSMP monitoring data |
| % willing HHs that bought an LLIN in year 3 of project | 2.75% |  |
| % potential HHs buy LLINs per year (Y1, Y2, Y3, Y4, Y5 post-project) |  |  |
| - Scenario 1 (2x increase in % from final project year to Y1, Y2, Y3 post-project, then remains constant) | 5.5%, 11%, 22% 22%, 22% |  |
| - Scenario 2 (1.5x increase in % from final project year to Y1 post project, 2x increase in Y2 & Y3 post-project, then remains constant) | 4%, 8%, 16%, 16%, 16% |  |
| - Scenario 3 (Y1 post-project same as final project year; 1.5x increase in Y2, Y3 post-project, then remains constant) | 2.75%, 4%, 6%, 6%, 6% |  |
| - Scenario 4 (% remains same as final project year) | 2.75% (all years) |  |
| - Scenario 5 (% remains same as final project year) | 2.75% (all years) |  |
| **Costs to workplace partners** |  |  |
| LLIN costs to workplace partners in year 3 of project | $253,346 | Results of costing analysis |
| Cost of BCC activities (seminars etc) in year 3 of project | $43,460 | Results of costing analysis |
| Costs of other malaria prevention activities in year 3 of project | $150,962 | Results of costing analysis |
| Annual increase in workplace partner contributions (e.g. by new partners starting activities or existing partners increasing scale) |  |  |
| - Scenario 1 | 20% |  |
| - Scenario 2 | 15% |  |
| - Scenario 3 | 10% |  |
| - Scenario 4 | 0% |  |
| - Scenario 5 | 0% |  |
| **Domestic resource mobilisation** |  |  |
| Total DACF budget (2018 budget from Resource Mobilisation Strategy, inflated to 2019 USD) | $392,936,875 | Ghana DRM strategy [32] |
| Total local MMDAs in Ghana | 260 | Ghana Districts [45] |
| Total least poor districts (Ashanti, Western, GA) | 32 | PSMP DCE paper [27] |
| Average annual budget for project area | $48,361,462 |  |
| Proportion of DACF budget allocated to malaria (Y1, Y2, Y3, Y4, Y5 post-project) |  | Ghana DRM strategy [32] |
| - Scenario 1 | 0.5%, 0.5%, 0.5%, 0.75%, 1.0% |  |
| - Scenario 2 | 0.5% (all years) |  |
| - Scenario 3 | 0%, 0%, 0.25%, 0.5%, 0.5% |  |
| - Scenario 4 | 0% (all years) |  |
| - Scenario 5 | 0% (all years) |  |
| Donor support to GMF |  |  |
| - Donor support to GMF Secretariat (1 FTE manager/administrator) | $25,000 | Annual project admin salary |
| - Match funding programmes (Y3 post-project; scenarios 1-4) | $750,000 | Ghana DRM strategy [32] |
| Private investments & fundraising for GMF (scenarios 1-4) |  |  |
| - Match funding programmes (Y3 post-project) | $750,000 | Ghana DRM strategy [32] |
| - Corporate fundraising (start Y3 post-project) | $50,000 | Ghana DRM strategy [32] |
| - Private investments (philanthropists, diaspora) (start Y3 post-project) | $50,000 | Ghana DRM strategy [32] |
| Annual increase in GMF contributions (for scenarios 1-4) |  |  |
| - Scenario 1 | 10% |  |
| - Scenario 2 | 5% |  |
| - Scenario 3 | 2.5% |  |
| - Scenario 4 | 0% |  |
| - Scenario 5 | (no donations) |  |
| *Abbreviations: BCC = behaviour change communication; DACF = district assembly common funds (district-level budget to promote local economic development); DCE = discrete choice experiment; DRM = domestic resource mobilisation; FTE = full time equivalent; GA = Greater Accra; GMF = Ghana Malaria Foundation; HH = household; LLIN = long-lasting insecticidal net; MMDA = metropolitan, municipal and district assemblies; Y = year* | | |
