# Supplementary material for: Using donor funding to catalyse investment in malaria prevention in Ghana: an analysis of the potential impact on public and private sector expenditure
Source: Malar J. 2022 Jun 27;21:203. doi: 10.1186/s12936-022-04218-2 (PMC9235193; doi:10.1186/s12936-022-04218-2)
Supplement: Supplementary file 2 — Additional file 2. Allocation of advocacy activities between (i) supporting workplace partnerships and (ii) broader domestic resource mobilisation activities (2019 USD). [file 12936_2022_4218_MOESM2_ESM.docx]

Additional File 2: Allocation of advocacy activities between (i) supporting workplace partnerships and (ii) broader domestic resource mobilisation activities (2019 USD)

|  | 2019 USD | | | |
| --- | --- | --- | --- | --- |
|  | Year 1 (July 2016 –  June 17) | Year 2 (July 2017 - June 18) | Year 3 (July 18 –  June 19) | Total |
| **ADVOCACY – supporting workplace partnerships** | | | | |
| **PERSONNEL** | 30,388 | 71,985 | 78,122 | 180,494 |
| **TRANSPORT** | 3,353 | 12,210 | 14,105 | 29,667 |
| **OTHER** | 54,638 | 157,902 | 53,101 | 265,641 |
| Venue hire/conference packages | 2,830 | 23,561 | 16,778 | 43,169 |
| Agency charges | 30,639 | 75,893 | 17,252 | 123,783 |
| Printing promotional materials | 15,356 | 23,230 | 13,185 | 51,770 |
| Media costs | 2,566 | 30,458 | 976 | 34,000 |
| LLINs | 0 | 0 | 0 | 0 |
| Other | 0 | 0 | 0 | 0 |
| Overheads | 3,248 | 4,760 | 4,911 | 12,918 |
| **TOTAL** | **88,379** | **242,096** | **145,327** | **475,802** |
| **ADVOCACY - resource mobilisation strategy support** | | | | |
| **PERSONNEL** | 26,899 | 62,542 | 46,317 | 135,759 |
| **TRANSPORT** | 2,011 | 11,565 | 15,287 | 28,863 |
| **OTHER** | 21,159 | 57,487 | 162,709 | 241,355 |
| Venue hire/conference packages | - | - | - | - |
| Agency charges | - | - | 120,647 | 120,647 |
| Printing promotional materials | - | - | - | - |
| Media costs | - | - | - | - |
| LLINs | - | - | - | - |
| Other | - | - | - | - |
| Overheads | 21,159 | 57,487 | 42,062 | 120,707 |
| **TOTAL** | **50,069** | **131,594** | **224,313** | **405,976** |
