# Supplementary material for: Using donor funding to catalyse investment in malaria prevention in Ghana: an analysis of the potential impact on public and private sector expenditure
Source: Malar J. 2022 Jun 27;21:203. doi: 10.1186/s12936-022-04218-2 (PMC9235193; doi:10.1186/s12936-022-04218-2)
Supplement: Supplementary file 3 — Additional file 3: Share of purchase costs of LLINs distributed through workplace partners over the three years of project implementation (2019 USD). [file 12936_2022_4218_MOESM3_ESM.docx]

# Additional File 3: Share of purchase costs of LLINs distributed through workplace partners over the three years of project implementation (2019 USD).

*
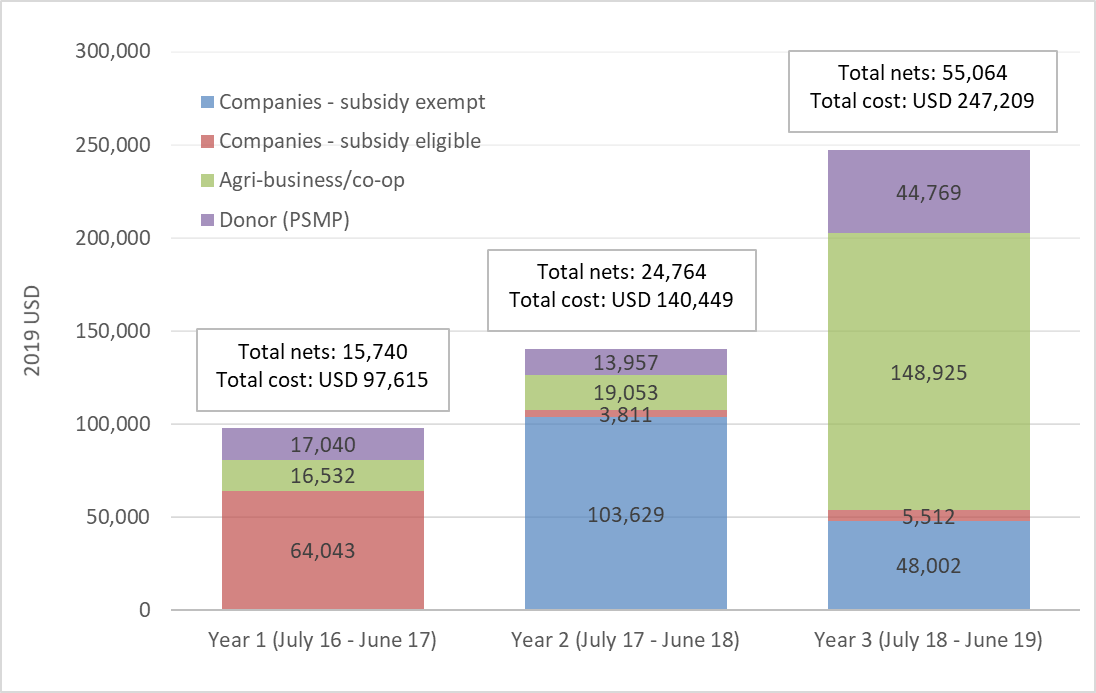
*

*NOTE: Private companies were eligible for subsidies of up to 20% and agricultural co-operatives for up to 40% for the first year of their participation; companies in the oil, extractive industries and financial sectors were not eligible for a subsidy.*
