# Supplementary material for: Using donor funding to catalyse investment in malaria prevention in Ghana: an analysis of the potential impact on public and private sector expenditure
Source: Malar J. 2022 Jun 27;21:203. doi: 10.1186/s12936-022-04218-2 (PMC9235193; doi:10.1186/s12936-022-04218-2)
Supplement: Supplementary file 4 — Additional file 4. Annualised donor investment costs. [file 12936_2022_4218_MOESM4_ESM.docx]

Additional File 4: Annualised donor investment costs

|  | Year 1 (July 16 - June 17) | | Year 2 (July 17 - June 18) | | Year 3 (July 18 - June 19) | | Total (2019 USD) | |
| --- | --- | --- | --- | --- | --- | --- | --- | --- |
|  | Actual cost (2019 USD) | Annualised (3+5 years) | Actual cost (2019 USD) | Annualised (2+5 years) | Actual cost (2019 USD) | Annualised (1+5 years) | Actual donor cost | Annualised donor cost |
| **SUPPORTING THE RETAIL SECTOR** |  |  |  |  |  |  |  |  |
| PSMP salaries & per diems | 179,742 | 25,605 | 189,007 | 30,337 | 172,081 | 31,766 | 540,831 | 87,708 |
| Overheads | 78,616 | 11,199 | 97,958 | 15,723 | 76,548 | 14,131 | 253,122 | 41,053 |
| Domestic transport | 5,366 | 764 | 16,993 | 2,728 | 40,607 | 7,496 | 62,967 | 10,988 |
| International transport | 13,779 | 1,963 | 19,885 | 3,192 | 15,317 | 2,827 | 48,980 | 7,982 |
| Market analysis | 36,626 | 5,218 | 95,014 | 15,250 | 73,622 | 13,590 | 205,263 | 34,058 |
| Stakeholder engagement & workshops | 29,228 | 4,164 | 5,344 | 858 | 11,610 | 2,143 | 46,182 | 7,165 |
| Generic marketing campaign | 0 | 0 | 0 | 0 | 303,228 | 55,975 | 303,228 | 55,975 |
| LLINs | 0 | 0 | 0 | 0 | 150,442 | 27,771 | 150,442 | 27,771 |
| **SUPPORTING WORKPLACE PARTNERSHIPS** |  |  |  |  |  |  |  |  |
| PSMP salaries & per diems | 175,355 | 24,980 | 262,273 | 42,096 | 195,673 | 36,121 | 633,301 | 103,198 |
| Overheads | 39,884 | 5,682 | 48,454 | 7,777 | 30,994 | 5,721 | 119,332 | 19,180 |
| Domestic transport | 34,977 | 4,983 | 57,114 | 9,167 | 36,648 | 6,765 | 128,739 | 20,915 |
| International transport | 0 | 0 | 0 | 0 | 0 | 0 | 0 | 0 |
| Partner engagement & workshops | 11,641 | 1,658 | 25,722 | 4,129 | 16,778 | 3,097 | 54,141 | 8,884 |
| Advocacy activities (awards ceremonies, media coverage) | 34,871 | 4,968 | 106,589 | 17,108 | 22,359 | 4,127 | 163,820 | 26,203 |
| LLINs | 0 | 0 | 82,442 | 13,233 | 0 | 0 | 82,442 | 13,233 |
| Promotional materials (brochures, factsheets) | 15,356 | 2,188 | 23,230 | 3,728 | 13,185 | 2,434 | 51,770 | 8,350 |
| **ADVOCACY FOR RESOURCE MOBILISATION** |  |  |  |  |  |  |  |  |
| PSMP salaries & per diems | 26,899 | 3,832 | 62,542 | 10,038 | 46,317 | 8,550 | 135,759 | 22,420 |
| Overheads | 21,159 | 3,014 | 57,487 | 9,227 | 42,062 | 7,765 | 120,707 | 20,006 |
| Resource Mobilisation Strategy development | 2,002 | 285 | 13,838 | 2,221 | 137,821 | 25,441 | 153,661 | 27,948 |
| **MANAGEMENT & CO-ORDINATION** | 319,410 | 45,502 | 361,155 | 57,968 | 483,745 | 89,298 | 1,164,310 | 192,768 |
| **Grand Total** |  |  |  |  |  |  | **4,418,996** | **735,805** |
